# Inhibition of growth of Asian keloid cells with human umbilical cord Wharton’s jelly stem cell-conditioned medium

**DOI:** 10.1186/s13287-020-01609-7

**Published:** 2020-02-21

**Authors:** Subramanian Arjunan, Shu Uin Gan, Mahesh Choolani, Vaishnevi Raj, Jane Lim, Arijit Biswas, Ariff Bongso, Chui Yee Fong

**Affiliations:** 1 Department of Obstetrics and Gynaecology, Yong Loo Lin School of Medicine, National University Health System, National University of Singapore, Kent Ridge, 119228 Singapore; 2 Department of Surgery, Kent Ridge, 119228 Singapore; 3 Department of Medicine, Yong Loo Lin School of Medicine, National University Health System, National University of Singapore, Kent Ridge, 119228 Singapore

**Keywords:** Characterization, Cell inhibition, Human Wharton’s jelly stem cell-conditioned medium, Keloid, SCID mice, Keloid tumor volume and weight

## Abstract

**Background:**

Keloid formation occurs in Caucasian, African, and Asian populations and is a severe psychosocial burden on patients. There is no permanent treatment for this problem as its pathogenesis is not properly understood. Furthermore, differences in keloid behavior between ethnic groups are not known. It has been hypothesized that keloids behave like benign tumors because of their uncontrolled growth. The present study evaluated the tumoricidal properties of human Wharton’s jelly stem cell-conditioned medium (hWJSC-CM) on fresh Asian keloid cells (AKCs).

**Methods:**

Human Wharton’s jelly stem cells (hWJSCs) and AKCs were isolated based on our previous methods. hWJSCs and human skin fibroblasts (HSF) (controls) were used to collect hWJSC-CM and HSF-conditioned medium (HSF-CM). AKCs were treated with hWJSC-CM and HSF-CM in vitro and in vivo in a human keloid xenograft SCID mouse model. The inhibitory effect of hWJSC-CM on AKCs was tested in vitro using various assays and in vivo for attenuation/abrogation of AKC tumors created in a xenograft mouse model.

**Results:**

qRT-PCR analysis showed that the genes FN1, MMP1, and VCAN were significantly upregulated in AKCs and ANXA1, ASPN, IGFBP7, LGALS1, and PTN downregulated. AKCs exposed to hWJSC-CM in vitro showed significant decreases in cell viability and proliferation, increases in Annexin V-FITC+ cell numbers, interruptions of the cell cycle at Sub-G1 and G2/M phases, altered CD marker expression, downregulated anti-apoptotic-related genes, and upregulated pro-apoptotic and autophagy-related genes compared to controls. When AKCs were administered together with hWJSC-CM into immunodeficient mice there were no keloid tumors formed in 7 mice (*n* = 10) compared to the untreated control mice. When hWJSC-CM was injected directly into keloid tumors created in mice there were significant reductions in keloid tumor volumes and weights in 30 days.

**Conclusions:**

hWJSC-CM inhibited the growth of AKCs in vitro and in xenograft mice, and it may be a potential novel treatment for keloids in the human. The specific molecule(s) in hWJSC-CM that induce the anti-keloid effect need to be identified, characterized, and tested separately in larger preclinical and clinical studies.

## Background

Keloids are dermal tumors categorized by a group of unusual fibroblasts with excessive deposition of extracellular matrix components such as collagen, elastin, fibronectin, and proteoglycans. Clinically, keloids are characterized by a painful pruritic raised scar that grows beyond the boundary of the original margin of wounds. It commonly involves the shoulders, ear lobes, upper arm, and anterior back [1]. It has been claimed that a total of 100 million patients develop scars in the developed world alone each year as a result of about 55 million elective and 25 million post-trauma operations [2]. Hypertrophic scars are similar to keloids but possess some clinical, histological, and epidemiological differences between them that suggest that they may be two distinct entities. Hypertrophic scars unlike keloids do not extend beyond the initial site of injury, have low recurrence rates after excision, and histologically possess well organized, wavy type III collagen bundles oriented parallel to the epidermal surface with abundant nodules containing myofibroblasts. Keloids on the other hand possess disorganized, large thick, types I and III hypo-cellular collagen bundles with no myofibroblast nodules. Keloids are poorly vascularized with widely scattered dilated blood vessels [2]. It has been suggested that, given these differences between keloids and hypertrophic scars, there is a need for proper characterization of keloids between ethnicities with the recommendation of a specific set of signature markers for their reliable identification.

The pathogenesis of keloid formation is poorly understood and to date, there is no successful treatment. It has been reported that the important obstacle to successful elimination was the lack of proper characterization of keloids in terms of its stemness nature and genetic properties [3]. Shih et al. [4] reported that a battery of 10 genes was significantly upregulated in keloid biopsy samples taken from the peripheral keloid-skin margin. It has been reported that the natural renewal of skin cells is under the control of mesenchymal stem cells (MSCs) and primary fibroblast-like cell populations obtained from various tissues contain mesenchymal progenitor and stem cells [5]. Moon et al. (2008) reported that the fibroblast cells derived specifically from keloid tissues were clearly multipotent stem cells that were being maintained in a proliferating and undifferentiated state by a definite native cytokine microenvironment in wounds [1]. Later, several independent research groups successfully isolated keloid-derived mesenchymal-like stem cells (KMLSCs) in adult keloid tissue which expressed the MSC CD markers CD13, CD29, CD44, CD90, fibronectin, and vimentin. KMLSCs could be differentiated into various lineages such as osteogenic, chondrogenic, adipocytic, smooth muscle, endothelial, and neuronal cell types.

The current treatments for keloids have been comprehensively reviewed, and it was concluded that none of them produced definite results. It was emphasized that there was an urgent need for developing novel therapies against keloids [2]. The available treatments include pressure therapy, silicone gel sheeting, cryotherapy, radiotherapy, laser therapy, surgical manipulation and administration of agents such as flavonoids, corticosteroids, interferons, and 5-fluorouracil. Interestingly, it has been reported that the treatment of murine skin defects with human MSCs results in scarless healing in 14 days [6].

We have studied a novel stem cell derived from the human umbilical cord Wharton’s jelly (hWJSCs) [7–12] that satisfies the criteria for MSCs recommended by the International Society of Cytotherapy [13]. Genomic, cell behavior, and stemness characterization studies showed that these hWJSCs lying in the gelatinous Wharton’s jelly within the umbilical cord are primitive and have uniquely different properties from bone marrow and other MSCs probably attributed to their embryological migration from the yolk sac and aorta-gonadal mesonephros (AGM) of the early developing human embryo [14]. Being protected within the umbilical cord, these primitive hWJSCs are not exposed to the insults of the adult environment.

Several groups including ours have reported that hWJSCs have unique tumoricidal properties [15–17]. Their transcriptome profiles also revealed a high expression of tumor suppressor and pro-apoptotic genes [18]. Extracts of hWJSCs [conditioned medium (hWJSC-CM) and cell lysates (hWJSC-CL)] inhibited the growth of breast and ovarian adenocarcinoma and osteosarcoma cells in vitro and attenuated or abolished the growth of breast tumors in xenograft animal models [15–17]. Furthermore, abdominal hernias in newborn infants have been successfully treated by attaching the infant’s own umbilical cord containing the Wharton’s jelly to the hernia with no ensuing scar and keloid formation [19, 20] probably due to the tumoricidal properties of the hWJSCs within the umbilical cord.

Given the tumoricidal properties of hWJSCs and the fact that keloids behave like benign tumors we wanted to evaluate the anti-keloid properties of hWJSCs. As a first step, we characterized AKCs to find out whether they are different from African and Caucasian samples and then tested the inhibitory effects of hWJSC-CM in vitro and in vivo on AKCs via various cell death assays and in vivo in a human keloid xenograft SCID mouse model.

## Methods

### Derivation of Asian keloid cells

Asian keloid cells (AKCs) were isolated based on a previously published protocol after obtaining written informed patient consent and ethical approval from the Institutional Domain Specific Review Board (DSRB), Singapore [21]. Briefly, human keloid tissues were collected from patients in the hospital and transported to the laboratory in a sterile vessel containing Hank’s balanced salt solution (HBSS, Invitrogen Life Technologies, Carlsbad, CA, USA). The keloid tissue was cut into 2 to 3 cm pieces and washed with sterile HBSS then incubated overnight at 4 °C with 3 mg/ml dispase (Invitrogen Life Technologies, Carlsbad, CA, USA). After overnight incubation, the epidermis was manually removed and the dermis was minced (1-mm3) into small pieces and incubated with 4 mg/ml collagenase type I at 37 °C in a 5% CO2 for 2 h. After enzymatic digestion, inactivated enzymes with DMEM low glucose (Invitrogen Life Technologies, Carslbad, CA, USA) contain 10% fetal bovine serum (FBS) (Biochrom AG, Berlin, Germany) and the cell suspension was filtered through 70 μm cell strainer [Becton Dickinson (BD), USA], centrifuged at 300×*g* for 5 min, supernatant discarded, and the cells were cultured in minimum essential medium (MEM) supplemented with 10% FBS, 100 U/ml Penicillin, 100 μg/ml streptomycin, 2 mM L-glutamine, 100 mM NEAA, and 550 μM 2-Mercaptoethanol (Invitrogen Life Technologies, Carlsbad, CA, USA) then seeded into a sterile 100 mm plastic tissue culture petri dish [Becton Dickinson (BD), USA] and incubated at 370 C in a 5% CO2. The morphology and growth of keloid cells were monitored and photographed under an inverted phase-contrast microscope.

### Human skin fibroblast cells

Commercial human skin fibroblast cells (HSFs) were purchased from ATCC (Manassas, USA) and cultured in DMEM high glucose (Invitrogen) with 10% FBS, 2 mM L-glutamine, and antibiotic-antimycotic mixture (Invitrogen), and then frozen for subsequent experiments.

### Derivation of human Wharton’s jelly stem cells

Human umbilical cords (UC) were obtained with informed patient consent and approval from the Ministry of Health, Domain Specific Review Board (DSRB) approval. The human Wharton’s jelly stem cells (hWJSCs) were derived from human umbilical cords according to a previously published protocol [22]. Briefly, the umbilical cord from each patient was transported to the laboratory in the transport medium (Hank’s balanced salt solution, HBSS, Invitrogen Life Technologies, Carlsbad, CA, USA). The UC was cut into smaller pieces (of about 1 cm long) and then cut open lengthwise. Without removing the umbilical blood vessels, each cut-open piece was placed with its inner surface face down into an enzymatic solution [2 mg/ml collagenase type I, 2 mg/ml collagenase type IV and 100 IU of hyaluronidase in DMEM medium (Invitrogen)] in 100 mm sterile plastic dishes (Becton Dickinson, BD, New Jersey, USA) and incubated at 37 °C in a 5% CO_2_-in-air atmosphere for 45 min to allow the Wharton’s jelly to slowly dissolve into the enzymatic solution. The enzymatic solution containing the Wharton’s jelly was then transferred to sterile 15 ml tubes (BD), syringed through an 18G needle to further break up the jelly to release the cells and centrifuged at 300 x g for 10 min. The supernatant was then decanted and the cell pellets were resuspended in a hWJSCs culture medium (complex) comprised of 80% DMEM high glucose supplemented with 20% FBS, 1% non-essential amino acids, 2 mM L-glutamine, 0.1 mM β-mercaptoethanol, 1% insulin-transferrin-selenium (ITS), antibiotic-antimycotic mixture (Invitrogen), and 16 ng/ml basic fibroblast growth factor (bFGF) (Millipore Bioscience Research Agents, Temecula, CA, USA).

### Preparation of hWJSC-conditioned and HSF-conditioned media

The hWJSCs and HSFs cell lines were separately cultured in T75 flasks in their respective culture media. When the cells were 70–80% confluent, the old medium was removed from each flask, washed with PBS and replaced with 10 ml of KOSR medium (DMEM-high glucose, 10% knockout serum replacement (KOSR), 1% L-glutamine, and 1% antibiotic-antimycotic mixture) and incubated for 72 h. After 72 h of growth of the cells in the KOSR medium, the medium was separated from the cells and called hWJSC conditioned medium (hWJSC-CM) and HSF conditioned medium (HSF-CM) respectively. Both hWJSC-CM and HSF-CM were diluted 1:1 v/v in KOSR medium and used as 50% hWJSC-CM and 50% HSF-CM for all experiments.

### Trypan blue vital counts

Asian keloid cells (AKCs) exposed to hWJSC-CM, HSF-CM, and control were quantified using trypan blue vital cell counts. An aliquot of the keloid cells was taken and stained with 0.4% Trypan Blue (vital dye) (Sigma) for 1 min at room temperature. The number of live cells (unstained) were counted using a hemocytometer (Hausser Scientific, Horsham, PA, USA).

### Cell viability (MTT) and cell proliferation (BrdU) assays

MTT: The cell viability assay was performed using a MTT reagent kit [3-(4, 5-dimethyl thiazolyl-2)-2, 5-diphenyltetrazolium bromide] according to the manufacturer’s instructions. Briefly, 10 μl MTT reagent (0.5 mg/ml) was added to 100 μl of medium bathing the cells in wells of tissue culture plates and the plates incubated for 4 h until a purple precipitate was visible. The medium was then removed and 100 μl of the detergent reagent was added into each well and incubation carried out in the dark for 2 h. Absorbance at 570 nm was spectrophotometrically measured using a microplate ELISA reader (μQuant, BioTek and Winooski, VT, USA) with a reference wavelength of 650 nm. BrdU: The cell proliferation assay was performed using a BrdU kit according to the manufacturer’s instructions (Cell Signaling, MA, USA). Briefly, the cells were incubated for 1–24 h with the BrdU solution followed by removal of the medium and incubation for 30 min with the fixing/denaturing solution. Subsequently, the cells were incubated for 1 h with BrdU detection antibody solution followed by three washes with the wash buffer. The HRP-conjugate solution was then added and incubated for 30 min followed by similar washing steps and incubation with the substrate solution for 30 min. The enzymatic reaction was finally stopped and the products quantified by measuring absorbance at 450 nm using a microplate ELISA reader.

### CD marker analysis

AKCs exposed to hWJSC-CM, HSF-CM, and control were dissociated and washed with PBS then blocked with 10% normal goat serum (NGS) (Invitrogen Life Technologies, Carlsbad, CA, USA) for 30 min to prevent non-specific binding. The cells were then incubated with primary antibodies (Biolegend, San Diego, CA, USA) for 1 h followed by incubation with Alexa Fluor®488 secondary antibody (Invitrogen, Carlsbad, CA, USA) for 30 min. The cells were analyzed using a CyAn™ ADP Analyzer (Beckman Coulter, Fullerton, CA, USA).

### Cell cycle analysis

Cell cycle analysis using flow cytometry of propidium iodide (PI) staining was done to compare the AKCs exposed to hWJSC-CM, HSF-CM, and control. Briefly, the cells were trypsinized and fixed with ice-cold 70% ethanol for 2 to 3 h. The fixed cells were washed with PBS and stained with 50 μg/ml PI in PBS containing 0.1% TritonX-100 and 50 μg/ml RNAse-A. Finally, the cells were filtered using a 70 μm nylon strainer to remove any cell clumps and then analyzed using a CyAn™ ADP Analyzer.

### Annexin V–FITC assay

The annexin V-FITC assay was carried out on the AKCs exposed to hWJSC-CM, HSF-CM, and control to evaluate rates of apoptosis at primary culture. Briefly, the cells were dissociated with TrypLE™ Express, washed once with PBS and then with Annexin V-binding buffer (1×). The cells were stained with 5 μl Annexin V-FITC at RT for 15 min and then counterstained with PI (1 μg/ml) and analyzed using a CyAn™ ADP Analyzer.

### Confocal microscopic analysis

The histological sections and adherent cells were fixed with 4% paraformaldehyde (Sigma) for 15 min and washed twice with PBS. The sections and cells were blocked with 10% normal goat serum (NGS) for 30 min and incubated with mouse primary monoclonal antibody Decorin (DCN) (5 μg/ml), mouse primary monoclonal antibody TGF-β1 (5 μg/ml), rabbit primary polyclonal antibodies fibromodulin (FMOD) (5 μg/ml), rabbit primary polyclonal antibodies TGF-β3 (5 μg/ml, Abcam, Cambridge, MA, USA), and human nuclear antigen (HNA) primary antibodies for overnight at 4 °C. This was followed by incubation with goat anti-mouse and goat anti-rabbit secondary antibodies (Alexa Fluor, Invitrogen) for 1 h. The sections and cells were then washed with PBS, stained with 4′-6-diamidino-2-phenylindole (DAPI; 0.5 μg/ml) (Invitrogen) for 5 min at room temperature (RT) and photographed using a confocal microscope (Olympus, Tokyo, Japan).

### Quantitative real-time polymerase chain reaction

Total RNA from all samples was extracted using the RNeasy Mini kit (Qiagen, Venlo, Netherlands). RNA samples were transcribed to cDNA using the Tetro cDNA Synthesis kit (Bioline, Eveleigh NSW, Australia). Primer sequences were taken from earlier published studies (Table 1). Quantitative real-time polymerase chain reaction (qRT-PCR) analysis was performed using ABI PRISM 7500 Fast Real-Time PCR System (Applied Biosystems, Waltham, MA, USA) using SYBR green master mix (Applied Biosystems) and relative quantification was performed using the comparative CT (2-ΔΔCT) method. The results were expressed as mean ± SEM from three replicates for individual experiments.

**Table 1:** Primers used for quantitative reverse transcription-polymerase chain reaction analysis

Gene name	Gene symbol	Gene ID	RefSeq ID
Glyceraldehyde-3-phosphate dehydrogenase	GAPDH	2597	NM_002046
Fibronectin 1	FN1	2335	NM_002026
Alpha-2-macroglobulin	A2M	2	NM_000014
Versican	VCAN	1462	NM_004385
Decorin	DCN	1634	NM_001920
Hypoxia-inducible factor 1 subunit alpha	HIF1A	3091	NM_001243084
Inhibin subunit beta A	INHBA	3624	NM_002192
Matrix metallopeptidase 1	MMP1	4312	NM_001145938
TNF alpha-induced protein 6	TNFAIP6	7130	NM_007115
Aggrecan	ACAN	176	NM_001135
Annexin A1	ANXA1	301	NM_000700
Insulin-like growth factor-binding protein 7	IGFBP7	3490	NM_001253835
Galectin 1	LGALS1	3956	NM_002305
Pleiotrophin	PTN	5764	NM_002825
Asporin	ASPN	54,829	NM_001193335
Serpin family H member 1	SERPINH1	871	NM_001207014
Neuronal regeneration-related protein	C5orf13/NREP	9315	NM_001142474
Transforming growth factor beta 1	TGFB1	7040	NM_000660
Transforming growth factor beta 3	TGFB3	7043	NM_003239
Baculoviral IAP repeat-containing 5	BIRC5	332	NM_001012270
Beclin 1	BECN1	8678	NM_003766
Autophagy related 5	ATG5	9474	NM_004849
Autophagy related 7	ATG7	10,533	NM_001136031
BCL2 associated X	BAX	581	NM_004324

### Effects of hWJSC-CM on human keloid tumors in xenograft SCID mice

In vivo studies were conducted on severely combined immunodeficient (SCID) mice, and all animal procedures were carried out in accordance with the National University of Singapore institutional animal guidelines (IACUC). Two protocols were used with the following experimental designs. *Combined protocol*: the combined protocol involved the administration of AKCs together with hWJSC-CM into mice to observe whether the hWJSC-CM prevented the formation of keloid tumors in the mice was based on previously published protocols [16, 17]. A total of 30 SCID mice were divided into 3 groups and the following agents administered subcutaneously into 2 injection sites, one site on each hind limb of each animal. Group 1: AKCs with hydroxyapatite (HA, 40 mg) (4 × 10^6^ cells), group 2: AKCs with hydroxyapatite (HA) (4 × 10^6^ cells) + HSF-CM (100 μl), and group 3: AKCs with hydroxyapatite (HA) (4 × 10^6^ cells) + hWJSC-CM (100 μl). HA was used to facilitate keloid tumor formation as in most studies HA is used in combination with tumorigenic agents to generate tumors rapidly [21]. *Separate protocol:* the separated protocol differed from the combined protocol where keloid tumors were first created in mice and respective treatments administered into the tumors to assess keloid attenuation or abolishment. Briefly, AKCs (4 × 10^6^ cells) were injected together with HA subcutaneously into both hind limbs of SCID mice and the mice monitored closely for tumor development. Our pilot studies showed that such keloid tumors created in mice were small in size (0.3 to 0.6 cm in diameter) and were not able to hold doses of 100 μl of conditioned medium, as there were spillage and rupture of the tumors after injection intra-tumorally. We therefore administered 50 μl per tumor intra-tumorally as this dose was sufficient to provide high concentrations of the hWJSC-conditioned medium in situ. Our main objective was to only see if the hWJSC-conditioned medium attenuated or abolished the growth of the keloid tumors. Thus, when tumors reached a size of 0.3–0.6 cm in diameter we administered 50 μl per tumor intra-tumorally under the following treatment groups. Group 1: PBS (50 μl), group 2: HSF-CM (50 μl), and group 3: hWJSC-CM (50 μl). Each arm had 9 SCID mice (*n* = 9). The control and experimental animals from all the above groups were sacrificed at the end of the study after 30 days using an inhalational overdose of carbon dioxide. Keloid tumors were collected for subsequent studies.

### Statistical analysis

Statistically significant differences between the treatment and control arms for all evaluations were carried out using one-way ANOVA with Bonferroni’s multiple comparisons post hoc analysis using the statistical package for Social Sciences (SPSS 13). The results were expressed as mean ± SEM from three replicates for three individual experiments, and a value of *p* < 0.05 was considered statistically significant.

## Results

### qRT-PCR and confocal microscopic analysis of Asian keloid cells

**Fig. 1:**
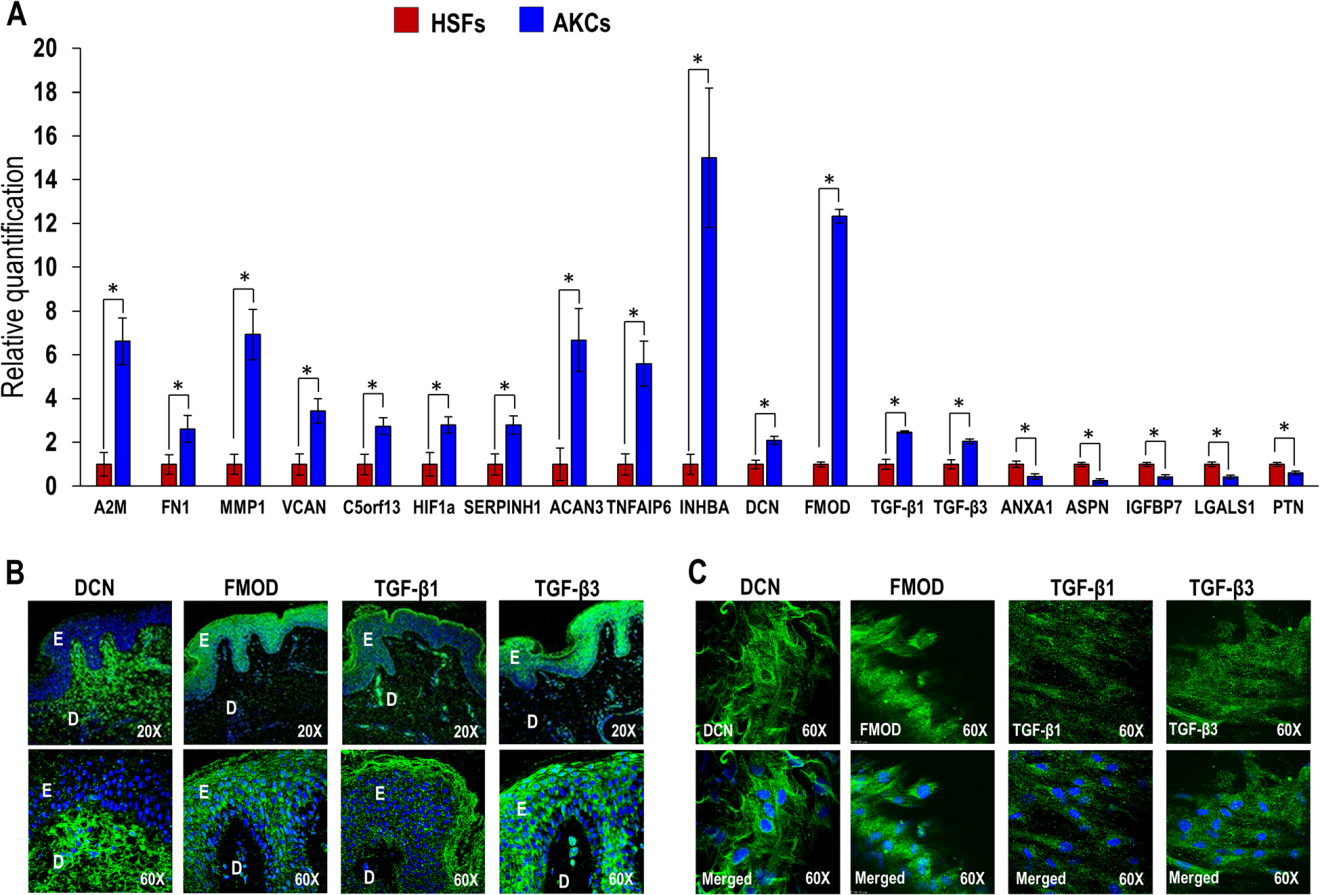
**a** qRT-PCR analysis of keloid-related genes (A2M, FN1, MMP1, VCAN, C5orf13, HIF1a, SERPINH1, ACAN3, TNFAIP6, INHBA, DCN, FMOD, TGF-β1, TGF-β3, ANXA1, ASPN, IGFBP7, LGALS1, and PTN). Confocal analysis of **b** paraffin section of keloid tissue and **c** AKCs adherent cells. All values are represented as mean ± SEM of at least three independent experiments. *p* < 0.05 was considered as statistically significant

Asian keloid cells (AKCs) were significantly upregulated for the keloid and matrix assembly-related genes such as A2M, FN1, MMP1, VCAN, C5orf13, HIF1a, SERPINH1, ACAN3, TNFAIP6, INHBA, DCN, FMOD, TGF-β1, and TGF-β3; and downregulated for ANXA1, ASPN, IGFBP7, LGALS1, and PTN compared to human skin fibroblasts (HSFs). The fold increases in expression levels for all these genes ranged from 2.10 to 17.58 and the fold increases for each gene were statistically significant compared to the HSFs (Fig. 1a). In situ cross-sections of keloid tissue and the monolayer cells derived from keloid tissue were positive for matrix assembly-related markers including DCN, FMOD, TGF-β1, and TGF-β3 (Fig. 1b, c).

### Cell morphology and Trypan blue live cell counts

**Fig. 2:**
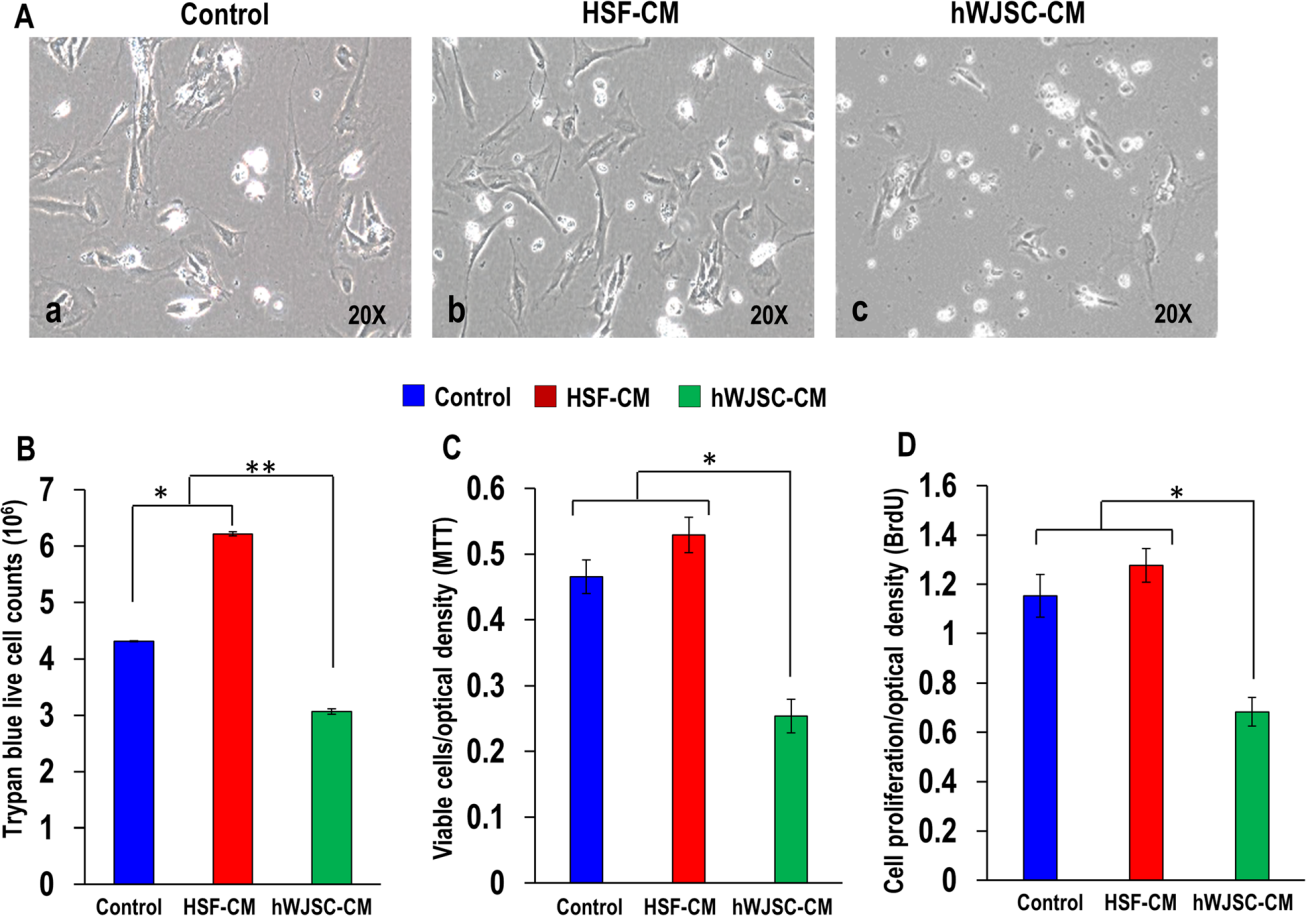
**a** Phase-contrast inverted optical images of AKCs grown in keloid medium (control), HSF-CM and hWJSC-CM. **b** Viable AKCs cell counts assessed by Trypan blue staining for AKCs grown in keloid medium (control), HSF-CM and hWJSC-CM. Assesment of **c** cell viability (MTT) and **d** cell proliferation (BrdU) of AKCs grown in keloid medium (control), HSF-CM and hWJSC-CM at day 3. All values are represented as mean ± SEM of at least three independent experiments. *p* < 0.05 was considered as statistically significant

AKCs cell numbers were decreased with lesser mitotic cells when exposed to hWJSC-CM (Fig. 2a(c)) compared to AKCs cultured in the presence of KOSR medium alone (untreated control) or human skin fibroblast conditioned medium (HSF-CM) where the cells continued their proliferation (Fig. 2a(a, b)). Trypan blue live cell counts showed that the live cell numbers for the AKCs cultured in HSF-CM were proliferative and significantly greater compared to untreated controls (Fig. 2b). However, the live cell numbers for AKCs cultured in hWJSC-CM were significantly lower (3.06 ± 0.05 × 10^6^) than the AKCs cultured in HSF-CM (6.21 ± 0.03 × 10^6^) and controls (4.30 ± 0.01 × 10^6^) (Fig. 2b).

### Cell viability (MTT) and cell proliferation (BrdU) assays

When the MTT assay for cell viability was used, the number of viable AKCs exposed to hWJSC-CM was significantly decreased on day 3 compared to HSF-CM and untreated controls (Fig. 2c). The mean ± SEM cell viability rates of AKCs were 0.25 ± 0.02% compared to 0.52 ± 0.02% for HSF-CM and 0.46 ± 0.02% for untreated controls. The BrdU assay for cell proliferation showed that the cell proliferation rates for the AKCs exposed to hWJSC-CM were significantly decreased on day 3 compared to HSF-CM and untreated controls (Fig. 2d). The mean ± SEM cell proliferation rates were 0.68 ± 0.05 for hWJSC-CM, 1.27 ± 0.06 for HSF-CM, and 1.15 ± 0.08 for controls.

### CD marker analysis

**Fig. 3:**
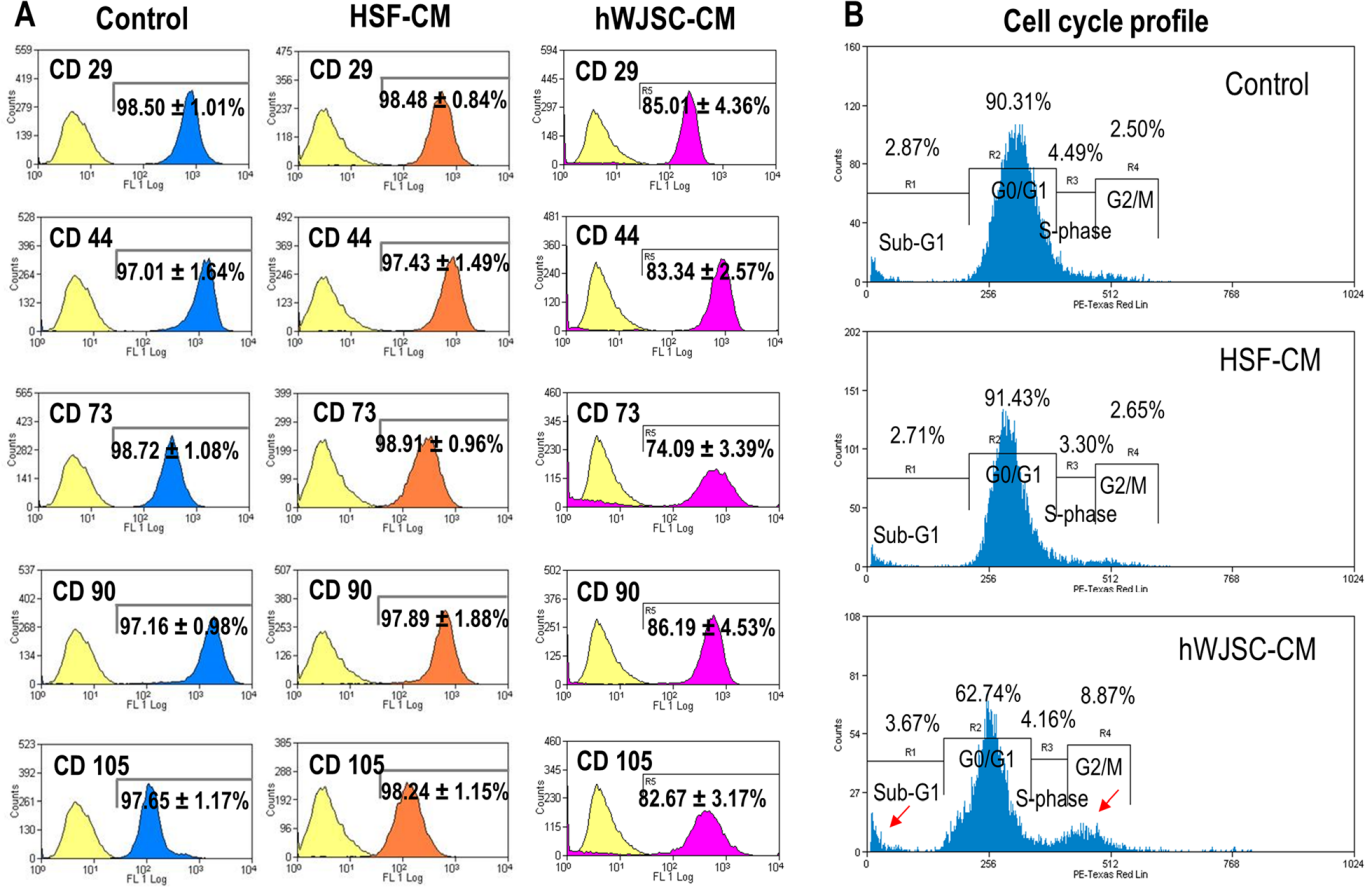
**a** Flow cytometry analysis of MSC CD marker profile for AKCs cultured in keloid medium (control), HSF-CM, and hWJSC-CM. **b** Cell cycle analysis of AKCs cultured in keloid medium (control), HSF-CM, and hWJSC-CM. All values are represented as mean ± SEM of at least three independent experiments. *p* < 0.05 was considered as statistically significant

Flow cytometry analysis showed the CD signature profile of AKCs was positive for CD29, CD44, CD73, CD90, and CD105. The CD marker levels were significantly decreased when exposed to hWJSC-CM compared to HSF-CM and controls. The percentage values for CD29, CD44, CD73, CD90, and CD105 were 98.50 ± 1.01%, 97.01 ± 1.64%, 98.72 ± 1.08%, 97.16 ± 0.98%, and 97.65 ± 1.17%, respectively, for the control group, 98.48 ± 0.84%, 97.43 ± 1.49%, 98.91 ± 0.96%, 97.89 ± 1.88%, and 98.24 ± 1.15%, respectively, for the HSF-CM group and 85.01 ± 4.36%, 83.34 ± 2.57%, 74.09 ± 3.39%, 86.19 ± 4.53%, and 82.67 ± 3.17%, respectively, for the hWJSC-CM group (Fig. 3a).

### Cell cycle analysis

AKCs cultured in KOSR medium (untreated control) and HSF-CM showed normal cell cycle profiles whereas the AKCs cultured in hWJSC-CM showed increased peaks in the Sub-G1 phase. The percentages were 2.87% for control, 2.71% for HSF-CM, and 3.76% for hWJSC-CM at the Sub-G1 phase. However, there were significantly high percentages of cells in the R4 (G2/M) phase (8.87%) in AKCs exposed to hWJSC-CM indicating that there were interruptions of the cell cycle at G2/M phase (Fig. 3b).

### Annexin V-FITC analysis for apoptosis

**Fig. 4:**
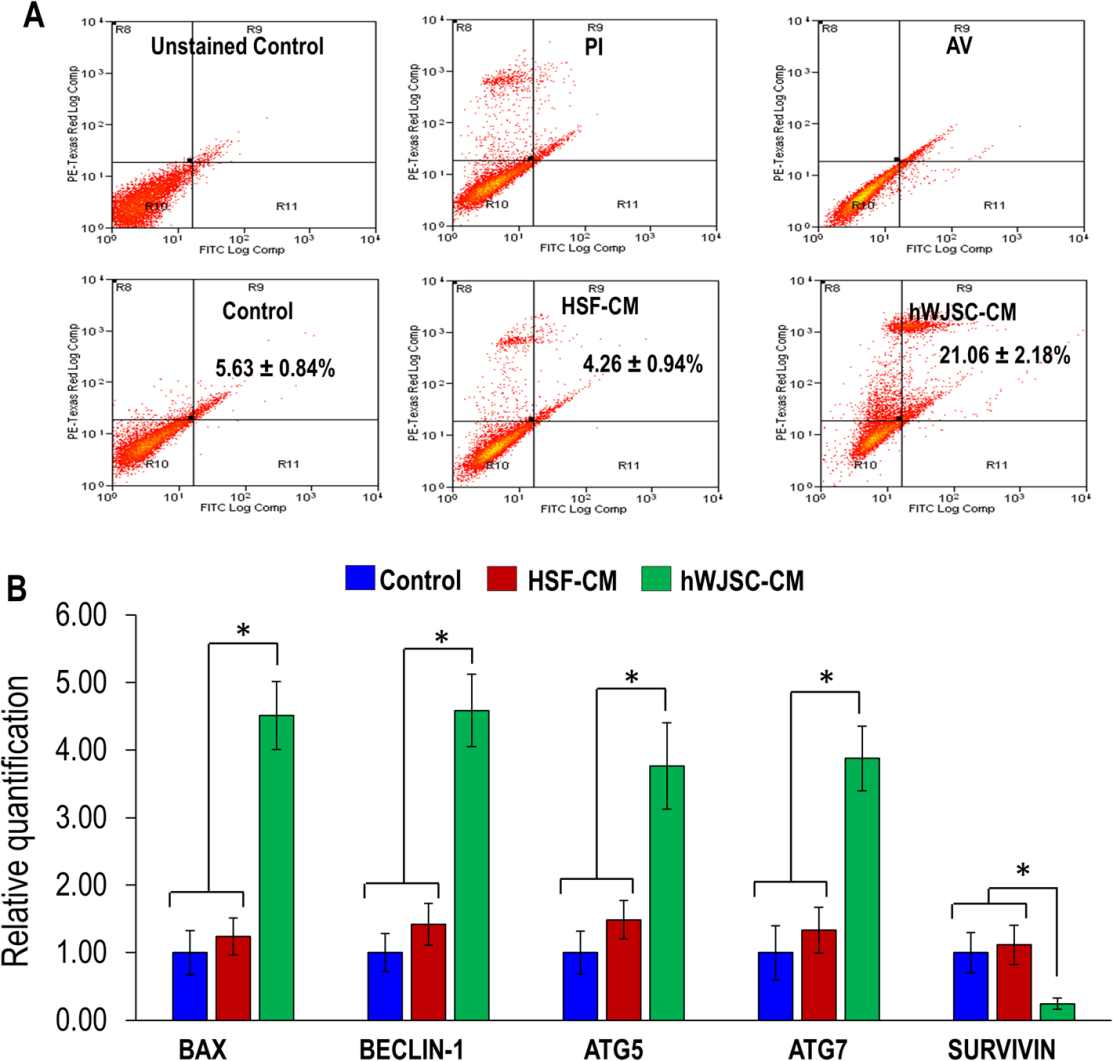
**a** Annexin-V analysis of AKCs grown in keloid medium (control), HSF-CM, and hWJSC-CM. **b** qRT-PCR analysis of pro-apoptotic, anti-apoptotic, and autophagy-related genes (BAX, SURVIVIN, BECLIN-1, ATG5, and ATG7) for AKCs grown in keloid medium (control), HSF-CM, and hWJSC-CM. All values are represented as mean ± SEM of at least three independent experiments. *p* < 0.05 was considered as statistically significant

AKCs cultured in hWJSC-CM were highly positive for Annexin V-FITC (21.06 ± 2.18) compared to controls (5.63 ± 0.84) and HSF-CM (4.26 ± 0.94). These mean increases in Annexin V-FITC-positive cell numbers were statistically significant (Fig. 4a). Furthermore, the anti-apoptotic-related gene (SURVIVIN) was downregulated and the pro-apoptotic and autophagy-related genes (BAX, ATG5, ATG7, and BECLIN-1) were upregulated in the AKCs exposed to hWJSC-CM compared to controls and HSF-CM. The fold decreases in expression levels for the anti-apoptotic-related genes ranged from 0.14 to 0.35 and the fold increases in expression levels for pro-apoptotic and autophagy-related genes ranged from 2.9 to 5.10. The fold increases for each gene were statistically significant compared to controls and HSF-CM (Fig. 4b).

### qRT-PCR of keloid genes exposed to hWJSC-CM

**Fig. 5:**
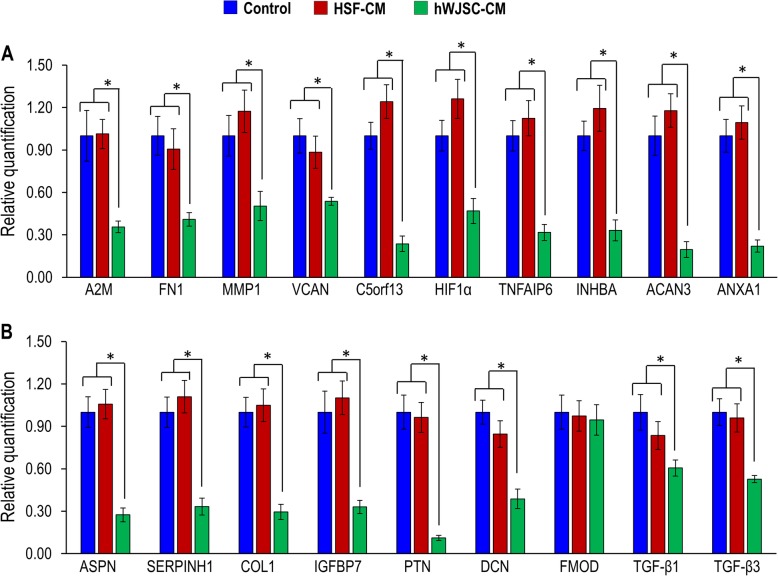
**a**, **b** qRT-PCR analysis of keloid and matrix assembly-related genes (A2M, FN1, MMP1, VCAN, C5orf13, HIF1a, TNFAIP6, INHBA, ACAN3, ANXA1, ASPN, SERPINH1, COL1, IGFBP7, PTN, DCN, FMOD, TGF-β1, and TGF-β3) for AKCs cultured in keloid medium (control), HSF-CM, and hWJSC-CM. All values are represented as mean ± SEM of at least three independent experiments. *p* < 0.05 was considered as statistically significant

The real-time polymerase chain reaction (qRT-PCR) results showed that the AKCs exposed to hWJSC-CM had significantly decreased keloid-related gene expression for A2M, FN1, MMP1, VCAN, C5orf13, HIF1a, TNFAIP6, INHBA ACAN3, ANXA1, ASPN, SERPINH1, COL1, IGFBP7, PTN, DCN, FMOD, TGF-β1, and TGF-β3 genes compared to AKCs exposed to untreated controls and HSF-CM. The fold decreases in expression levels for all these genes ranged from 0.06 to 0.55. The fold decreases for each gene were statistically significant compared to HSF-CM and controls (Fig. 5a, b).

### Effects of hWJSC-CM on human keloid tumors in xenograft SCID mice

#### Combined protocol

**Fig. 6:**
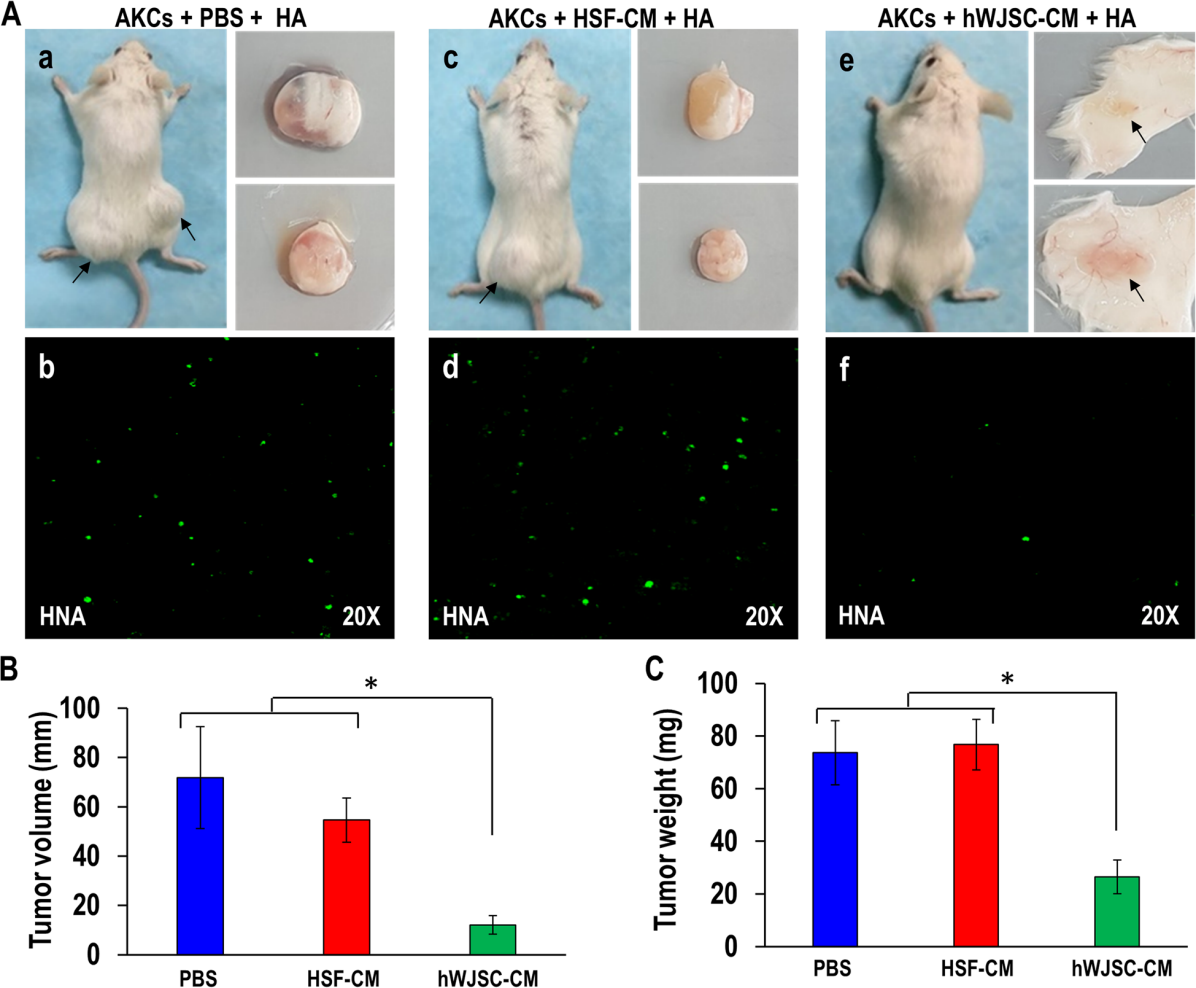
**a** Combined protocol (The administration of AKCs together with hWJSC-CM into SCID mice) of keloid xenograft SCID mice. **a**(a, c, and e) AKCs + HA, AKCs + HA + HSF-CM, and AKCs + HA + hWJSC-CM were injected into subcutaneous sites (both hind limbs) per animal (*n* = 10). **a**(b, d, and f) Human nuclear antigen (HNA) staining of keloid tumor from AKCs + HA, AKCs + HA + HSF-CM, and AKCs + HA + hWJSC-CM. **b** Evaluation of keloid tumor volume and **c** tumor weight. All values are represented as mean ± SEM of at least three independent experiments. *p* < 0.05 was considered as statistically significant

When AKCs were administered together with hWJSC-CM + hydroxyapatite, (HA) into 10 immunodeficient mice subcutaneously (combined protocol), there was no keloid tumor formation in 7 mice and 3 small lumps in the remaining 3 mice after 30 days (HA is conventionally administered to facilitate tumor formation) (Fig. 6a(e)). This was in comparison to keloid tumor formation that was observed in all the animals in the untreated control group [AKCs + HA (10 mice)] and HSF-CM + HA group (10 mice) (Fig. 6a(a, c)). Human nuclear antigen (HNA) staining for AKCs in the tumors and injection sites showed a greater number of HNA positive cells in the untreated control and HSF-CM groups compared to the hWJSC-CM + HA group (Fig. 6a(b, d, f)). Keloid tumor volumes (Fig. 6b) and tumor weights (Fig. 6c) were significantly reduced in the hWJSC-CM treatment group compared to untreated control and HSF-CM groups. The mean ± SEM keloid tumor volumes after 30 days were 71.78 ± 20.67 mm for untreated controls, 54.65 ± 8.97 mm for HSF-CM, and 12.04 ± 3.69 mm for hWJSC-CM. The mean ± SEM keloid tumor weights after 30 days were 73.70 ± 12.12 mg for untreated controls, 76.70 ± 9.58 mg for HSF-CM, and 26.50 ± 6.38 mg for hWJSC-CM.

#### Separate protocol

**Fig. 7:**
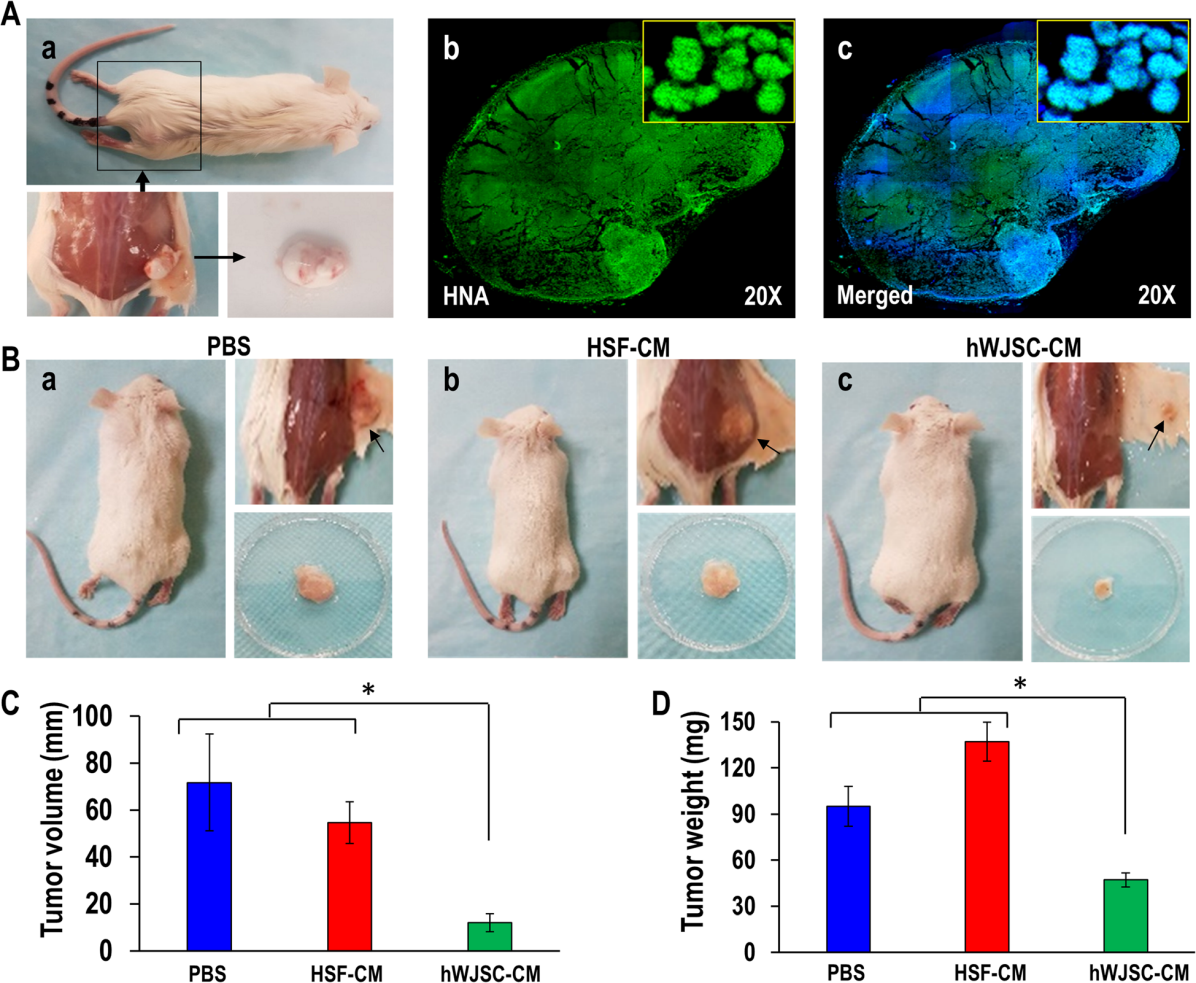
**a** Separate protocol (Keloid tumors were first created in SCID mice about the size of 0.3–6 cm in diameter and respective treatments administered through intra-tumorally) of keloid xenograft SCID mice. **a**(a) AKCs + HA were injected into subcutaneous sites to induce keloid tumors. **a**(b, c) Human nuclear antigen (HNA) staining of keloid tumor from AKCs + HA on day 7. **b**(a–c) PBS, HSF-CM, and hWJSC-CM treatments were administered intra-tumorally to each animal (*n* = 9). **c** Evaluation of keloid tumor volume and **d** tumor weight. All values are represented as mean ± SEM of at least three independent experiments. *p* < 0.05 was considered as statistically significant

When AKCs + HA was administered subcutaneously to the mice, keloid tumors were formed as early as 7 days in all animals, at all injection sites and in all three groups (30 mice, 60 tumors) (Fig. 7a(a)). Keloid tumor section (one mouse per group derived on day 7) showed positive for HNA staining, and these results confirmed that the keloid tumor developed from AKCs (Fig. 7a(b, c)).

Keloid tumors were completely abolished (absent) in 5 animals (10 tumors) and tumor shrinkage observed in 4 animals (8 tumors) after 23 days in the mice treated with hWJSC-CM (*n* = 9) compared to untreated controls (*n* = 9) and HSF-CM (*n* = 9) where none of the tumors were abolished or shrunken in size at 23 days (Fig. 7b(a–c)). Keloid tumor volumes (Fig. 7c) and tumor weights (Fig. 7d) were significantly reduced in the hWJSC-CM treatment group compared to untreated controls and HSF-CM. Mean ± SEM keloid tumor volumes were 90.52 ± 15.56 mm for untreated controls, 101.70 ± 11.97 mm for HSF-CM and 28.20 ± 4.13 mm for hWJSC-CM. Mean ± SEM keloid tumor weights were 95.14 ± 12.87 mg for untreated controls, 137.00 ± 12.71 mg for HSF-CM, and 47.11 ± 4.57 mg for hWJSC-CM at 23 days.

## Discussion

The present study evaluated novel biomarker expression on keloid samples from Asian patients in comparison with normal human skin fibroblasts. The results confirmed that 14 genes (A2M, FN1, MMP1, VCAN, C5ORF13, HIF1A, SERPINH1, ACAN3, TNFAIP6, INHBA, DCN, FMOD, TGF-β1, and TGF-β3) were upregulated and 6 genes (ANXA1, ASPN, COL1, IGFBP7, LGALS1, and PTN) were downregulated. The results of expression of some of the genes are consistent with those obtained from African and Caucasian samples by other workers [4, 23]. However, the results of the present study revealed for the first time the downregulation of five genes (ANXA1, ASPN, IGFBP7, LGALS1, and PTN) which is in contrast to the upregulation of the same genes in African and Caucasian keloid samples [4].

The upregulated 14 genes (A2M, FN1, MMP1, VCAN, C5ORF13, HIF1A, SERPINH1, ACAN3, TNFAIP6, INHBA, DCN, FMOD, TGF-β1, and TGF-β3) play important roles in keloid pathogenesis. FN1 is involved in cell adhesion and migration, wound healing, blood coagulation, host defense, and metastasis. Its upregulation has been linked to enhanced e*xtracellular matrix* (ECM) synthesis. VCAN is involved in cell adhesion, proliferation, migration, and angiogenesis, playing a crucial role in tissue morphogenesis and maintenance. ACAN and TNFAIP6 were found to interact with hyaluronic acid in keloid fibroblasts when upregulated [24] but are not well studied in keloid or skin [25]. Seifert et al. (2008) also found higher INHBA gene expression in keloid-derived fibroblasts compared to those from healthy skin. SERPINH1 has been suggested to stimulate excessive collagen deposition in keloids [4]. HIF-1 is a master regulator of cellular and systemic homeostatic responses to hypoxia while MMP1 is involved in ECM breakdown. INHBA gene is the regulator of G-protein signaling 4 and shown to be differentially altered in fibroblasts isolated from spatially distinct areas of the keloid [26]. DCN aids collagen matrix assembly and FMOD interacts with type I and II collagen fibrils, playing a role in ECM assembly. TGF-β1 regulates such cellular functions as cell growth, proliferation, differentiation, and apoptosis [27]. TGF-β3 helps regulate cellular adhesion and ECM formation [28].

Of the downregulated five genes (ANXA1, ASPN, IGFBP7, LGALS1, and PTN) observed in the present study, ASPN and C5ORF13 have been suggested to be negative regulators of TGF-*β*1. ASPN was shown to bind directly to TGF-*β*1 and C5ORF13 was shown to induce a non-fibrogenic myofibroblast-like phenotype in 3 T3 cells [29]. ANXA1 binds phospholipids, inhibits phospholipase A2, and has anti-inflammatory activity [30]. LGALS1 plays a role in cell-cell and cell-matrix interactions, and in tumor progression [4]. PTN is involved in cell growth, differentiation, and tumor progression. The higher-level expression of PTN in hypertrophic scars and the low-level expression in keloids suggest different pathomechanisms between keloids hypertrophic scars [31].

Several groups including our own have shown that hWJSCs have tumoricidal properties, inhibiting the growth of various cancers both in vitro and in vivo [15–17]. Our group showed in a microarray transcriptome profile study that hWJSCs have increased expression of tumor suppressor genes and anti-apoptotic genes compared to other stem cell types such as human embryonic stem cells and bone marrow mesenchymal stem cells [18]. We recently showed that hWJSC-CM inhibited the growth of lymphoma cells suggesting the presence of anti-cancer molecules secreted by the hWJSCs into the conditioned medium [32]. Other workers have reported that conditioned media prepared from adipose tissue-derived and bone marrow MSCs contained various anti-fibrotic factors [28, 33]. All these results allude to the fact that the hWJSC secretome may contain tumoricidal-like molecules that inhibit keloid cell growth given the fact that keloids behave like benign tumors with their uncontrolled growth.

The results of the present study revealed that cell viability and proliferation rates were significantly lower in fresh uncultured Asian keloid cells (AKCs) exposed to hWJSC-CM compared to controls. Although AKCs expressed CD29, CD44, CD73, CD90, and CD105 markers [1, 34], their expression was significantly reduced in the AKCs after exposure to hWJSC-CM suggesting that AKCs were losing their stemness properties but not in controls. AKCs exposed to hWJSC-CM appear to be inhibited via apoptosis as they were arrested at Sub-G1 and G2/M phases [33], which is the same focal point of cell arrest by other anti-leukemic agents [35]. Furthermore, we observed increased numbers of Annexin-V-positive cells in AKCs exposed to hWJSC-CM compared to controls. Additionally, the qRT-PCR results showed that the anti-apoptotic gene (SURVIVIN) was downregulated and pro-apoptotic and autophagy-related genes (BECLIN-1, BAX, ATG5, ATG7) were upregulated in AKCs exposed to hWJSC-CM, further confirming that the possible mechanisms by which hWJSC-CM induce AKCs cell death is through autophagy and apoptotic pathways. These results tally with our previous reports that hWJSC-CM increased oxidative stress, thus inducing apoptosis in lymphoma cells and keloids [32, 34].

Of note, is that this study also revealed the upregulation of certain genes that play a key role in wound healing such as TGF-β1, TGF-β3, and other ECM-related genes. TGF-β overexpression accelerates ECM deposition by increasing the synthesis of collagen, integrin, and fibronectin [28]. The results of the present study revealed that TGF-β1, TGF-β3, and collagen 1 genes were significantly decreased in AKCs when exposed to hWJSC-CM, suggesting that hWJSC-CM downregulated TGF-β1 and TGF-β3 activity and reduced ECM synthesis by decreasing the expression of collagen and fibronectin genes. Also, it has been reported that impaired angiogenesis is the cause of refractory wounds [36]. Studies have suggested that MSC-based therapies with platelet-rich plasma are able to promote wound healing by enhanced angiogenesis [37, 38]. Furthermore, the clinical studies reported by Cervelli et al. (2010) and Nicoli et al. (2013) have demonstrated that the use of a three-dimensional matrix of hyaluronic acid with platelet-rich plasma can enhance tissue proliferation and promotes wound healing with reduction of hypertrophic scars [39, 40]. Several groups, including our group, have shown that hWJSCs secrete high levels of hyaluronic acid, anti-inflammatory, and antifibrotic cytokines (IL10, VEGF, and HGF), and suppress the production of pro-inflammatory cytokines (TNF-α and IL-6) that may be responsible for reduced scarring to allow better healing [7, 34, 36].

The percentage induction of keloid tumors in the murine xenograft model of the present study is consistent with that of other workers [21, 41–46]. Our results showed that AKCs administered with hydroxyapatite (HA) produced 100% of keloid tumors in SCID mice and HNA staining confirmed that the tumors were indeed those of human origin. Interestingly, although hWJSC-CM did not abolish the growth of the keloid tumors completely there was a significant reduction in the keloid tumor volumes and weight in both the combined and separate protocols compared to controls. Perhaps, further reductions in keloid tumor growth may have taken place if the treatment doses were increased or the study prolonged for longer periods.

From an embryological point of view, several distinct compartments (amnion, subamnion, Wharton’s jelly, perivascular area) have been recognized in the human umbilical cord by several authors [9, 47, 48]. The stemness properties of cells between these compartments were shown to be distinctly different [9]. Unfortunately, some reports do not accurately specify the compartment from which their MSCs were derived and usually generalize or misname the derivation as from simply Wharton’s jelly. This makes meaningful comparisons of data between groups difficult.

Many groups have shown the anti-keloid effects of MSCs [33, 34, 49, 50], but one study showed that MSCs derived from the subamniotic membrane of the umbilical cord showed enhanced keloid fibroblast growth in vitro [51], with no confirmatory studies in in vivo preclinical animal models. Given the differences in stemness properties of cells between subamnion and Wharton’s jelly compartments [9, 52], it is possible that these different responses are due to differences in the secretome of the two cell types because of their different embryological development, cell form, and function. Additionally, the different ethnic sources of the keloid cells used in the studies may also account for the differences in anti-keloid behavior because the enhanced keloid cell growth by subamniotic membrane stem cells was observed in Caucasian samples while the keloid inhibitory effects observed with Wharton’s jelly stem cells was on Asian samples. Several studies have shown that hWJSCs secrete various factors such as interleukins, cell membrane proteins, cell adhesion molecules, cadherins, growth factors, hyaluronic acid, and glycosaminoglycans suggesting that they may be responsible for inhibiting the AKCs or keloid formation in the SCID mice [7, 34, 53]. It has been reported that the behavior of keloid-derived fibroblasts can be altered by MSCs through paracrine signaling [54, 55] which is a key mechanism of MSC therapeutic effects [56, 57].

## Conclusions

The present study suggests that hWJSC-CM inhibits the growth of AKCs in vitro and in vivo. It may thus be an attractive novel treatment for keloids in the human. One or more interesting anti-keloid molecules may exist in hWJSC-CM that need to be identified, characterized and further validated for anti-keloid growth in human clinical trials.

## Data Availability

Supporting data can be obtained from the corresponding author.

## Abbreviations

AGM: Aorta-gonadal mesonephros
AKC: Asian keloid cells
ATG: Autophagy
bFGF: Basic fibroblast growth factor
DCN: Decorin
DMEM: Dulbecco’s modified Eagle’s medium
ECM: Extracellular matrix
FBS: Fetal bovine serum
FN: Fibronectin
FMOD: fibromodulin
HNA: Human nuclear antigen
HSF: Human skin fibroblasts
HSF-CM: Human skin fibroblasts conditioned medium
hWJSCs: Human Wharton’s jelly stem cells
hWJSC-CM: Human Wharton’s jelly stem cells conditioned medium
HA: Hydroxyapatite
ITS: Insulin-transferrin-selenium
KOSR: Knockout serum replacement
MSC: Mesenchymal stem cells
NGS: Normal goat serum
qRT-PCR: Quantitative real-time polymerase chain reaction
SCID: Severely combined immunodeficient
TGF: Transforming growth factor-beta
VCAN: Versican
